# Combined Pre- and Postnatal Minimally Invasive Approach to Complicated Pulmonary Sequestrations

**DOI:** 10.1055/s-0040-1713901

**Published:** 2020-09-18

**Authors:** Martina Ichino, Francesco Macchini, Anna Morandi, Nicola Persico, Isabella Fabietti, Andrea Zanini, Ernesto Leva

**Affiliations:** 1 Department of Pediatric Surgery, Fondazione IRCCS Ca' Granda Ospedale Maggiore Policlinico di Milano, Milano, Lombardia, Italy; 2 Department of Obstetrics and Gynecology “L. Mangiagalli”, Fetal Medicine and Surgery Service, Fondazione IRCCS Ca’ Granda Ospedale Maggiore Policlinico di Milano, Milano, Lombardia, Italy; 3 Department of Clinical Science and Community Health, Università degli Studi di Milano, Milano, Lombardia, Italy

**Keywords:** pulmonary sequestration, fetal therapy, thoracoscopy, laser coagulation, congenital lung malformation

## Abstract

Pulmonary sequestration (PS) is mostly asymptomatic but there is a proportion of fetuses that develop hydrops, leading to fetal or neonatal death. Fetal treatments are available, but postnatal management of the residual lesions is not uniformly defined. We present two cases of combined pre- and postnatal minimally invasive approach to complicated extra-lobar PS.

Patient 1 presented with complicated PS at 31 weeks of gestation. Ultrasound-guided laser coagulation of the anomalous artery was successful. The patient was born asymptomatic at 38 weeks. Neonatal magnetic resonance imaging (MRI) showed a residual mass, confirmed by computed tomography (CT) at 6 months. No systemic artery was described, but perfusion was present. We decided for thoracoscopic resection. A residual artery was identified and sealed. Patient 2 presented with complicated PS at 25 weeks of gestation, underwent laser coagulation of the anomalous artery and was born asymptomatic at 38 weeks. Neonatal MRI showed persistence of the lesion, confirmed by CT scan at 4 months. We proceeded with thoracoscopic resection. A residual vessel was ligated. The patients 1 and 2 are now 24 and 21 months old, respectively, and healthy.

Prenatal treatment of complicated PS is a life-saving procedure.

Postnatal thoracoscopic resection of the residual lesion is feasible and safe; we believe it is the best course of treatment to grant the complete excision of the malformation.

## Introduction

Pulmonary sequestration (PS) is the second most common prenatally diagnosed congenital pulmonary airway malformation. 1 2 3 It consists of a mass of nonfunctioning pulmonary parenchyma, disconnected from the bronchial tree, and receiving an anomalous systemic arterial blood supply. PS can be classified as intralobar PS (IPS), when it is included in the lobar pleura and usually drains in the pulmonary venous system, and as extralobar PS (EPS), when the mass has its own pleura and systemic venous drainage. 2 Most of the PSs are asymptomatic during pregnancy but, in a minority of cases, PS can be complicated by pleural effusion, mediastinal shift, and eventually hydrops fetalis which is reported in 6.8 to 20% 1 4 of prenatally diagnosed PS cases. Hydrops is a harbinger of fetal demise or neonatal death if left untreated 1 5 6 7 ; overall mortality for nonimmune hydrops fetalis is reported between 76 and 78%. 8 9 Given the poor prognosis of PS complicated by hydrops, prenatal treatment with open fetal surgery has been attempted, but it results in high incidence of maternal complications and elevated risk of preterm labor. 10 With the progression of minimally invasive fetal surgical techniques, various treatments have been developed to increase the chance of survival of these fetuses and reduce impact on the mother. As with every technical progress, new questions come into light, the new approaches need verification and patients care must be adjusted accordingly. Data concerning the outcomes of prenatal treatment of PS are mostly represented by case reports and case series, 1 11 12 13 14 15 16 17 18 all reporting a very high rate of survival of prenatally treated fetuses compared with the poor prognosis of the fetuses affected by complicated PS that are left untreated. The most popular techniques are fetal thoracentesis, thoracoamniotic shunts, and ultrasound-guided laser ablation or sclerotherapy of the anomalous feeding vessel. 1 11 Standardized indications for fetal intervention in prenatally diagnosed PS still need to be defined, hydrops fetalis seems universally considered as an indication for fetal intervention, but case series also report intervention in case of severe pleural effusion. 1 5 14 Thoracentesis and thoracoamniotic shunts are symptomatic treatments that reduce the hydrothorax and consequently the mediastinal shift, reducing the compression on the mediastinal vessels and therefore the risk of fetal hydrops. Thoracentesis is, however, a temporary solution. Fluid often reaccumulates in the pleural space with possible need of multiple procedures. Cavoretto et al 11 report reaccumulation of the fluid in six out of six reviewed cases. Survival rate for patients with PS that were treated with thoracentesis is reported as 83%. 5 Thoracoamniotic shunt positioning could theoretically avoid the need for multiple thoracentesis; however, reaccumulation of fluid was reported by Cavoretto et al in 2 out of 17 reviewed cases, 11 and thoracoamniotic shunts may dislocate with possible increased morbidity. 19 A survival rate of 92% is reported by Witlox et al 5 after thoracoamniotic shunting for PS. The laser ablation and the sclerotherapy of the feeding vessel have the aim of reducing the blood sequestration from fetal circulation and the PS mass itself, thus treating the disease and reversing its complications. 12 Sclerotherapy has been reported as effective in four out of four reviewed cases 11 with a survival rate of 75% 5 and laser ablation was effective in 10 out of 10 reviewed cases 11 with a survival rate of 100%. 5 To the best of our knowledge, only one death after laser treatment for PS has been reported later on. 15 Moreover, Witlox et al 5 report a lower rate of respiratory failure at birth in cases treated with vascular laser ablation as compared with cases that underwent palliative treatments. Although complications, such as damage to adjacent organs, uterine or fetal bleeding, and placental abruption 14 must be expected, to the best of our knowledge, they have not been reported.

Given the rarity of prenatally complicated PS, these are still preliminary data and the best pre- and postnatal management of this malformation remains to be confirmed. The ablation of the anomalous vessel seems a safe and effective approach and is the technique adopted at our center. It is considered in the presence of a considerable risk of intrauterine demise or preterm delivery, such as in case of hydrops, tense polyhydramnios and significant Doppler abnormalities.

The management of the residual masses after birth is not uniformly defined at present: some surgeons remove the eventual remnant after birth, 11 others don't. 14 We present two cases of EPS that were treated with fetal laser coagulation of the anomalous vessel and were subsequently thoracoscopically removed with elective surgery after birth.

## Case Report

### Case 1

**Fig. 1:**
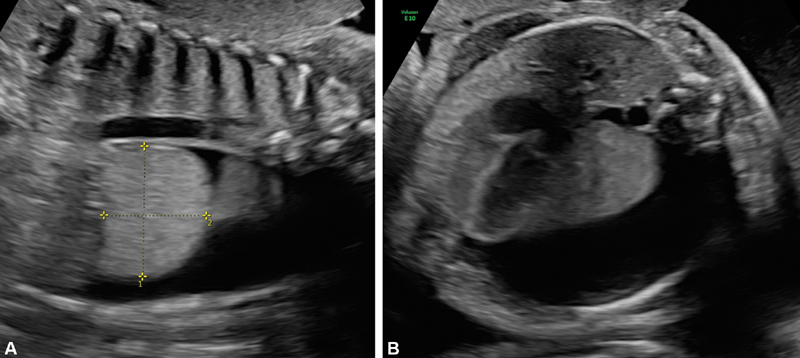
Prenatal images of patient described as case 1. Sagittal view of the echogenic mass in the inferior portion of the left fetal lung ( **A** ) and transverse view showing severe unilateral hydrothorax ( **B** ).

Case 1 is a male fetus that presented with left inferior pulmonary malformation consistent with EPS at 31 weeks of gestation. Obstetric ultrasound showed severe unilateral hydrothorax occupying >50% of the thoracic cavity, with right mediastinal shift and subcutaneous edema. An echogenic mass in the inferior portion of the left fetal lung, measuring 45 mm × 37 mm × 38 mm, was observed ( Fig. 1 ) and Power Doppler demonstrated blood supply through a main feeding vessel. Based on these findings, the option of fetal intervention by ultrasound-guided laser coagulation of the anomalous vessel was discussed with the parents and, after informed consent, the procedure was performed at 31 ^3/7^ weeks of gestation. Briefly, after administration of fetal intramuscular anesthesia and maternal local analgesia, a 17-G needle (Cook Medical, Bloomington, Indianapolis, Indiana United States) was introduced under ultrasound guidance through the fetal thorax to reach a distance of few millimeters from the feeding vessel. Subsequently, a 400-µm diameter diode laser fiber (Dornier MedTech GmbH, Wessling, Germany) was advanced through the sheath of the needle. Laser energy with a power of 15 to 20 Watts was applied for few seconds twice to thrice to ensure complete cessation of blood flow. Aspiration of 50 mL of thoracic fluid was performed. The complete procedure lasted 30 minutes, and there were no intraoperative complications. Serial follow-up ultrasound examinations showed a normal fetal growth and the Doppler and a progressive improvement of the pleural effusion until complete resolution by 34 weeks of gestation. The size of the EPS was significantly reduced to 21 mm × 19 mm × 18 mm at 38 weeks.

**Fig. 2:**
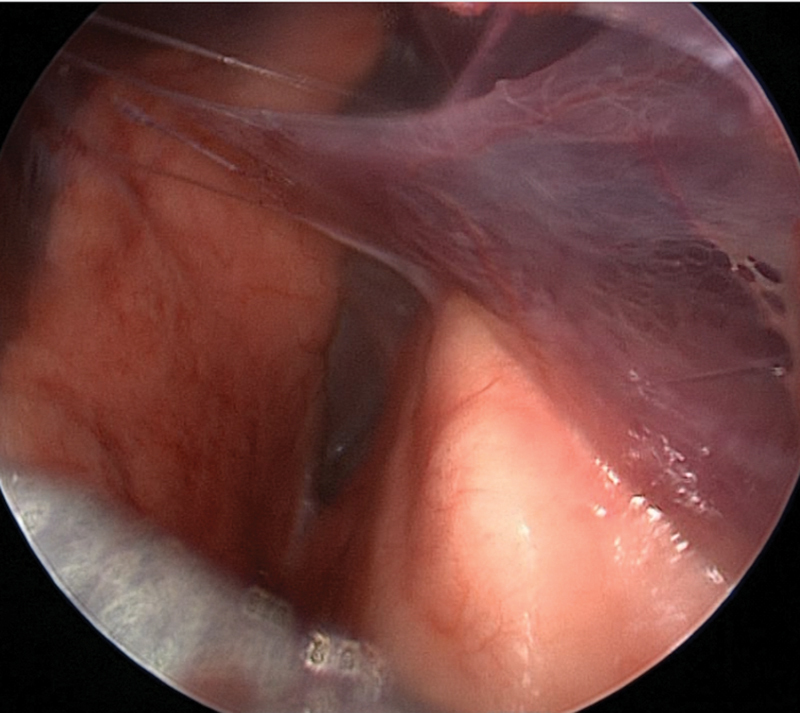
Presence of numerous adhesions between the visceral and the parietal pleura in the patient described as case 1.

The newborn was delivered vaginally at 38 ^5/7^ weeks of gestation with a birth weight of 3,295 g and he was asymptomatic throughout the postnatal period. Given the prenatal diagnosis of PS, despite prenatal treatment, the patient was managed according to our internal protocol for pulmonary airways malformations. The protocol foresees chest X-ray at birth to exclude possible acute complications (pneumothorax and massive mediastinal shift), chest magnetic resonance imaging (MRI) in spontaneous sleep in the first month of life to confirm diagnosis without additional radiation, and without need for sedation, and preoperative chest computed tomography (CT) scan with contrast. The chest X-ray showed normal radiological findings and a neonatal MRI in spontaneous sleep was performed and showed persistence of a solid mass in the left costofrenic angle with diameters of 17 mm × 11 mm × 20 mm, with a suspicion of a cystic component. No anomalous vessel was visualized by MRI. Due to the persistence of the malformation, a clinical follow-up was started. Echocardiography ruled-out anatomical or functional heart disease. A preoperative chest CT scan with contrast was planned and performed at 6 months of age. It showed the mass with 12 mm × 9 mm dimension and no visible afferent vessel, but perfusion inside the mass was present. Decision was made to proceed with surgery. At 7 months of age the patient underwent thoracoscopy, he was positioned in right lateral decubitus and a 3-mm trocar was inserted under the tip of the scapula. Capnothorax was created at 5 mm Hg at 1L/min. Two additional trocars were inserted on the anterior axillary line: a 3-mm one in the third to fourth intercostal space and a 5-mm one in the fifth to sixth. At the inspection of the thoracic cavity, numerous adhesions were evident between the visceral and the parietal pleura ( Fig. 2 ). After lysis of the adhesions with Just-Right Sealing System (3 mm), the PS was identified and confirmed as extralobar. It presented strong adhesions to the left inferior lobe. An apparently patent feeding vessel coming from the thoracic aorta was identified as well. The vessel was closed proximally with a Hem-o-lock clip and sealed distally with Just-Right Sealing System and divided. Histology confirmed the PS.

The postoperative course was uneventful with no need for mechanical ventilation. Chest drain was removed on postoperative day 2 and the patient was discharged on the postoperative day 3. The patient is now 24-month old, he presents a regular growth and is in good health with no respiratory symptoms.

### Case 2

**Fig. 3:**
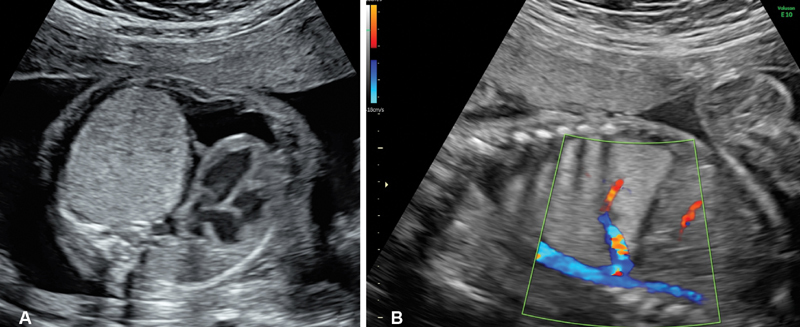
Prenatal images of patient described as case 2. Transverse view of the fetal thorax illustrating a giant solid lung mass with massive mediastinal shift ( **A** ) and sagittal view of the thorax showing a systemic feeding artery arising from the aorta ( **B** ).

The second case is a male fetus with diagnosis of left inferior PS at 25 weeks of gestation. Ultrasonographic follow-up evidenced progressive worsening of mediastinal shift and unilateral hydrothorax ( Fig. 3 ), development of ascites, and cardiac failure. The fetal MRI confirmed a 30 mm × 37 mm × 44 mm mass with an anomalous systemic vessel of 2.6 mm of diameter originating from the aorta at the thoracoabdominal passage, consistent with an EPS. Concomitant hydrothorax with maximum width of 16 mm was described, as well as right mediastinal shift and mild ascites. Given the presence of hydrops, a fetal treatment with ultrasound-guided laser coagulation of the anomalous vessel was offered, and after parental consent the procedure was performed at 26 ^1/7^ weeks of gestation. The procedure was performed using the same technique as described for case 1 with a total duration of 24 minutes. No intraoperative or postoperative complications were observed, and there was progressive resolution of fetal hydrops by 30 weeks of gestation. A repeat fetal MRI performed at 30 ^4/7^ weeks of GA showed a reduction in size of the malformation (22 mm × 24 mm × 19 mm), no visible vessels and only minimal residual hydrothorax. No further complications presented during pregnancy.

**Fig. 4:**
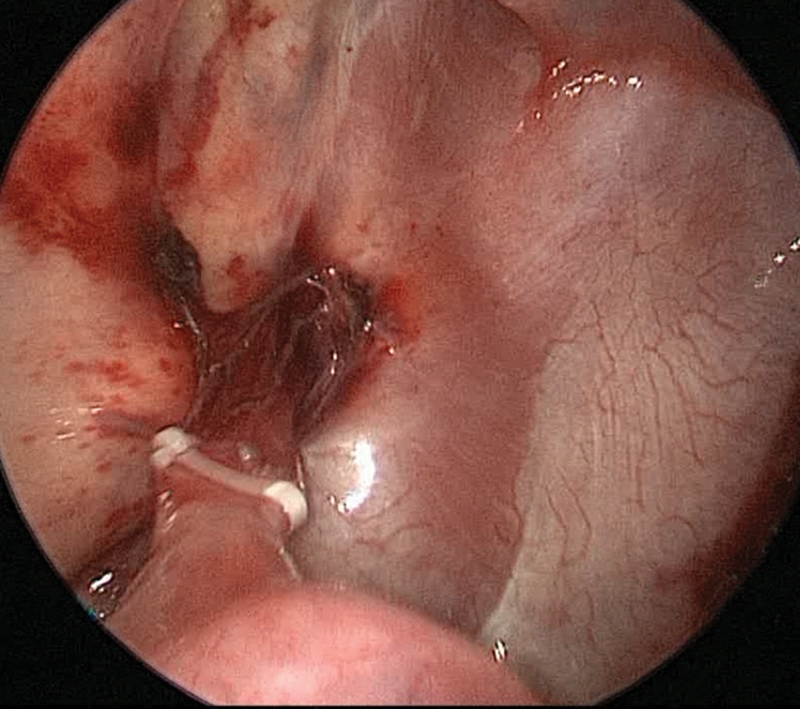
Residual systemic vessel in the patient described as case 2, closed with Hem-o-Lock clip before division with Just-Right 5-mm stapler.

The newborn was delivered vaginally, asymptomatic, at 38 ^5/7^ weeks of gestation with a birth weight of 4,070 g. Chest X-ray at birth was normal. Neonatal chest MRI showed persistence of a 19 mm × 19 mm × 21 mm mass in the left supradiaphragmatic paravertebral region. An echocardiography ruled-out anatomical or functional heart disease so a clinical follow-up was started. A chest CT scan was performed at 4 months of age. The malformation was described of 16 mm × 12 mm, adherent to the costovertebral pleura and with adhesions to the diaphragmatic pleura. No systemic vessel was visualized. Decision was made to proceed with surgery. At 5 months of age the patient underwent thoracoscopic resection of the mass with the same technique as for the previous case. Again, at the inspection of the pleural cavity severe adhesions were evident. The sequestration was confirmed as extralobar and was strongly attached to the left inferior lobe. A residual systemic vessel was identified, closed with a Hem-o-lock clip and divided with Just-Right 5-mm stapler, given the vessel dimension ( Fig. 4 ). Histology confirmed a PS with necrotic-calcified areas of neoangiogenesis. No mechanical ventilation was required postoperatively. The postoperative course was characterized by mild bleeding from the chest drain treated conservatively. The chest drain was removed on the postoperative day 3 and the patient was discharged on the postoperative day 4.

The patient is now 21-month old with regular growth and no respiratory symptoms.

## Discussion

The high mortality of prenatally complicated PS is well known. 1 5 6 7 8 9 20 Nowadays, the availability of fetal surgery has opened a variety of treatment opportunities that has changed the outcome of these severe forms. Ultrasound-guided laser ablation of the anomalous vessel seems to grant the most effective and lasting results, 5 and it can be said with enough confidence that it is safe and effective in the short term, and that the benefits of this procedure outweigh its possible complications such as damage to adjacent organs, uterine or fetal bleeding, placental abruption, and premature birth. 14 However, the postnatal management of these lesions remains to be debated. While it is evident that prenatally treated PS that show symptoms at birth need to be removed, the optimal treatment course of the asymptomatic residual lesions is not clear.

Oepkes et al 12 in 2007 reported a case of laser coagulation of the anomalous artery of a PS. The procedure was successful and lead to an asymptomatic newborn. Nonetheless, a CT scan at 1.5 years of age showed persistence of the sequestration with systemic blood supply. The parents declined surgery because of the absence of symptoms. A second report of this procedure was made by Ruano et al, 13 the procedure was uncomplicated and reversed the fetal hydrops, but 7 weeks after the procedure, blood flow reappeared in the anomalous vessel. The patient underwent lobectomy on the 15th day of life because of onset of respiratory distress. Cavoretto et al 11 presented a series of eight patients with complete resolution in three while five patients required postnatal sequestrectomy for persistence of the lesion at imaging. Cruz-Martinez et al 14 reported another series of eight cases describing a complete interruption of vascular flow with one procedure in five cases, while three cases required a second laser treatment. Postnatal CT scan showed a small residual non perfused mass in all cases. Given the absence of vascular flow, sequestrectomy was not performed. Mallmann et al 18 reported five cases of laser ablation for EPS. Three cases required one intervention, and in two cases, the procedure was repeated to obtain complete flow interruption. In four cases complete regression was observed and one case required sequestrectomy after birth.

In both our cases, the fetal procedure was successful, leading to regression of fetal symptoms and to a subsequently uneventful pregnancy. Both patients were asymptomatic at birth, but a residual mass was present. In one case, the mass presented signs of revascularization at CT scan. In the other case, the mass persisted without signs of reperfusion, but presenting fibrous connection to the pleura and the diaphragm. In both cases, we believed the indication for sequestrectomy was appropriate given the persistence of the lesion and their characteristics. Moreover, given the experience of our center with thoracoscopic surgery, the possibility to offer the sequestrectomy with a minimally invasive approach granted a reduced surgical impact. The presence of pleural adhesions as a result of prenatal treatment, did not impede the minimally invasive approach. As we evidenced intraoperatively, even if the postnatal CT didn't show the anomalous systemic vessel, the vascular structure was still present. As we previously discussed, the revascularization of the anomalous vessel after laser coagulation has been described. 12 13 14 At present, it is not possible to predict the incidence of revascularization of the sequestration for the lack of long-term data. Given the absence of follow-up studies, especially regarding the cases treated conservatively after birth, it is not possible to strongly support one choice over the other. Nonetheless, we believe that if the PS persists after birth, it should be treated as a PS without prenatal treatment, and therefore should undergo elective thoracoscopic resection to avoid the development of symptoms. Especially if it shows signs of perfusion, if it seems intralobar, and therefore with a possibility of hybrid lesion, or if it causes compression on the healthy lung. Thoracoscopic sequestrectomy for PS, when performed by experienced surgeons, is a safe minimally invasive procedure even in the presence of adhesions following fetal treatment. The discussed elements lead us to favor, at present, postnatal surgery after prenatal laser coagulation. Moreover, we think that, in case of conservative approach, an adequate clinical and radiological follow-up has to be warranted to evidence a possible recurrence. In such cases, thoracic MRI can be considered as a primary imaging technique for the follow-up to reduce the radiation impact. 21

## Conclusion

Prenatal treatment of complicated PS is a life-saving procedure. It results in regression of symptoms, but not always disappearance, of the lesion. Postnatal thoracoscopic resection of the residual lesion is feasible and safe in trained hands; we believe that it is the best course of treatment to grant the complete excision of the malformation, thus avoiding the risk of future complications.

## Notes

Importance for a Pediatric Surgeon Pulmonary sequestrations can occasionally present with prenatal complication, correlated to high incidence of fetal or neonatal death. The availability of fetal therapies is changing the outcome of these complicated lesions, but post-natal treatment of the residual malformation remains debated. We present our experience of a combined pre- and postnatal approach to complicated pulmonary sequestration.
